# Dietary sources of energy and macronutrient intakes among Flemish preschoolers

**DOI:** 10.1186/0778-7367-69-5

**Published:** 2011-11-01

**Authors:** Willem De Keyzer, Yi Lin, Carine Vereecken, Lea Maes, Herman Van Oyen, Erika Vanhauwaert, Guy De Backer, Stefaan De Henauw, Inge Huybrechts

**Affiliations:** 1 Department of Nutrition and dietetics, University College Ghent, Gent, Belgium; 2 Department of Public Health, Ghent University, Ghent, Belgium; 3 Unit of Epidemiology, Scientific Institute of Public Health, Brussels, Belgium; 4 Flemish Institute for Health Promotion and disease prevention, Brussels, Belgium

## Abstract

This study aims to identify major food sources of energy and macronutrients among Flemish preschoolers as a basis for evaluating dietary guidelines. Three-day estimated diet records were collected from a representative sample of 696 Flemish preschoolers (2.5-6.5 years old; participation response rate: 50%). For 11 dietary constituents, the contribution of 57 food groups was computed by summing the amount provided by the food group for all individuals divided by the total intake of the respective nutrient for all individuals. Bread (12%), sweet snacks (12%), milk (6%), flavoured milk drinks (9%), and meat products (6%) were the top five energy contributors. Sweet snacks were among the top contributors to energy, total fat, all fatty acids, cholesterol, and complex and simple carbohydrates. Fruit juices and flavoured milk drinks are the main contributors to simple carbohydrates (respectively 14% and 18%). All principal food groups like water, bread and cereals, vegetables, fruit, milk and spreadable fats were under-consumed by more than 30% of the population, while the food groups that were over-consumed consisted only of low nutritious and high energy dense foods (sweet snacks, sugared drinks, fried potatoes, sauces and sweet spreads). From the major food sources and gaps in nutrient and food intakes, some recommendations to pursue the nutritional goals could be drawn: the intake of sweet snacks and sugar-rich drinks (incl. fruit juices) should be discouraged, while consumption of fruits, vegetables, water, bread and margarine on bread should be encouraged.

## Introduction

The diet in childhood is not only of great importance for the well-being and growth of the child, it is also a potential determinant of adult morbidity and mortality [1,2]. However, some important gaps in the diet of Flemish preschoolers have been identified in the past and have already been discussed in depth by comparing nutrient and food intakes of Flemish preschoolers with respectively the Belgian age-specific recommended dietary allowances (RDA) for nutrients [3] and the Flemish food-based dietary guidelines (FBDG) for preschoolers in Flanders [4]. In summary, more than half of the children did not comply with the water recommendation. Furthermore, intakes of saturated fatty acid (SFA), mono-unsaturated fatty acid (MUFA) and poly-unsaturated fatty acid (PUFA) were not in line with the recommendations at population level. Therefore, the diet of Flemish preschoolers can be an important target for modifying the risk for cardiovascular disease (CVD) in later life [3]. When comparing the food group intakes among preschoolers in Flanders with the Flemish FBDG (Table 1), we found that for almost all food groups more than half of the children did not reach the minimum recommendations. However, the consumption of meat products and sugar and fat-rich products like soft drinks and biscuits exceeded the upper levels of the recommendations [4].

**Table 1:** Flemish Food Based Dietary Guidelines for preschoolers [23]

Food group	Children 1 - 3 years	Children 3 - 6 years
**Water**		
Liquids	0.5 - 1 litre	1.5 litre
**Cereal and potatoes**		
Bread	1-3 slices (30-100 g)	3-5 slices (100-150 g)
Potatoes	1-2 pieces (50-100 g)	1-4 pieces (50-200 g)
**Vegetables**		
Cooked vegetables should be alternated with raw vegetables	1-2 vegetable spoons (50-100 g)	2-3 vegetable spoons (100-150 g)
**Fruit**	1-2 pieces (100-200 g)	1-2 pieces (100-200 g)
**Dairy and calcium enriched soy drinks**		
Milk (whole fat milk up to the age of 4 years)	4 beakers (500 ml)	4 beakers (500 ml)
Cheese	1/2 slice (10 g)	1/2*-1 *slice (10-20 g)
**Meat, fish, eggs and meat substitutes (e.g. tofu)**		
Meat, meat products (cold cuts), poultry, fish (raw weight)	30-50 g	50-75 g
*OR*		
Meat substitute (tofu, tempe, mycoproteins)	30-50 g	50-75 g
*OR*		
Mushrooms (cooked weight)	50 g	100 g
*OR*		
Eggs	1 per week	1 per week
*OR*		
Legumes (dry weight)	1 tablespoon (15 g)	2 tablespoons (30 g)
Legumes (cooked weight)	3 tablespoons (50 g)	6 tablespoons (100 g)
**Fat (baking & spreadable)**		
On bread	5 g per slice of bread	5 g per slice of bread
Oil or baking fat	≤ 15 g	≤ 15 g
**Residual group (e.g. soft drinks, candy, etc.)**	discouraged	discouraged

The relative contribution of specific foods and food groups to total nutrient intakes has been studied since the early eighties, when a new concept of 'important' nutrient sources was introduced in addition to the concept of 'rich' nutrient sources [5]. Whereas rich sources were foods with the greatest concentration of a nutrient, important sources were foods that contributed most to a population's intake. Important sources of nutrients can be strongly influenced by 'nutrient density' of the food, its 'frequency of consumption' and the quantity consumed. Knowledge of such sources, combined with information about the degree to which people meet recommended nutrient intakes, is not only useful for recommending changes in food intakes to pursue nutritional goals [6], but also for the conceptualisation of new/revised dietary guidelines and dietary intake assessment instruments [7-9].

To date, no comprehensive analysis has been undertaken to identify energy and macronutrient sources in Flemish preschoolers. Therefore the current paper includes two important study aims. Firstly, it investigates the major food sources of macronutrients and energy among preschoolers in Flanders. Secondly, it investigates how changes in food intakes (while taking into account their nutrient contributions) could induce changes in nutrient intakes. Based upon those results, age-specific suggestions in the context of FBDG can be made in order to increase compliance of nutrient recommendations.

## Methods

The present study used data of the Flanders preschool dietary survey (data collected from October 2002 until February 2003), in which usual dietary intake was estimated from 3-day estimated dietary records (3d EDR), completed by the parents. To ensure that all days of the week would be equally covered in the dietary records, the days to be registered were determined beforehand. The sampling design and methods have been described in detail previously [10]. In brief, a random cluster sampling design at the level of schools, stratified by province and age was used (the age range for the target population were preschool children 2.5-6.5 years old). Also, the response rate and the representativeness of the study sample were discussed before (50% response rate and 49% after data-cleaning) [10]. Comparison of the study sample with the Flemish population confirmed good demographic representativeness of our study sample. Data on educational level of parents showed that, compared to the Flemish population, participants were higher educated. This was definitely true for those participants with good quality food diaries. The school headmasters, teachers and parents were informed about the study objectives, aims and dietary assessment methods during a school meeting. Oral and written instructions were provided for the recording of foods and drinks consumed by children. Portion sizes were described as natural units (like fruit), known amounts in grams or ml, or using household measures like 'a small glass' or 'half a plate'. Teachers were asked to report what the children consumed at school so that the parents/proxies could include it in the diaries. For the current analyses diaries containing insufficiently detailed descriptions of the food products and portion sizes consumed (e.g. when the parents did not include descriptors like 'low fat' or 'whole grain' in their diaries or when they did not include portion sizes (no standard units or grams)) were excluded. Thus, only good quality food diaries, including three completed record days were included (*n *= 696; 66% of collected diaries). Two dietitians, with long-standing experience in nutritional epidemiological fieldwork, performed this exclusion procedure of the EDR.

The percentage of underreporters was already described in depth in a previous paper and was shown to be low (< 2% of the children when using Goldberg cut-offs adapted for children) [3]. Underreporters have not been excluded from the study sample that was used for the present analyses.

The food composition data for calculating nutrients were based on the following tables: the Belgian food composition table NUBEL [11], the Dutch food composition database NEVO [12], the food composition table of the Belgian Institute Paul Lambin [13], and McCance and Widdowson's UK food composition table [14].

In this paper, energy and ten nutrients (nine macronutrients and cholesterol), that have public health significance in terms of meeting either nutrient requirements or dietary guidance, are reported. In total 936 foods and composite dishes were encoded in the original database. All recipes that were described in detail as ingredients in the diaries were encoded as ingredients in the original database. However, in order to classify foods easily into food groups of the Flemish FBDG, eight extra composite dishes had to be disaggregated (nasi goreng, nasi goreng with egg, spaghetti bolognaise, chicken ragout, turkey ragout, lasagna, macaroni ham/cheese sauce, and stew). Spaghetti bolognaise for instance was disaggregated into pasta, minced meat, onions, tomatoes, carrots and margarine (the source used for recipe description was the recipe list of the Flemish EPIC-soft version 2004) [15]. In addition to those recipes that needed to be disaggregated, the authors sometimes had to aggregate ingredients into their original mixed/complex food in order to allow comparison with the FBDG. For instance when parents reported the ingredients of homemade bread, this had to be aggregated into bread in order to facilitate comparison with the food groups of the FBDG. In total 116 food items were recomposed. After the aggregation and disaggregating procedures, food items were divided into 57 food groups of similar nutrient content, based on the classification of the Flemish FBDG and the expert opinion of the investigators (see food groups listed in tables 2 and 3). In the Flemish FBDG, products within a food group have been categorised into three groups: food items that are to be preferred -- the 'preference group' (e.g. fresh fruit), food items that should be consumed with moderation -- the 'moderate group' (e.g. fruit juice) and food items that should be avoided -- the 'residual group' (e.g. confectionery, soft drinks, ...).

**Table 2:** Contribution from all food groups to energy, fat, fatty acids and cholesterol (n = 696)

	Nutrients and dietary constituents														
	
	**Food intake**^**f**^			Energy		Total Fat		SFA		MUFA		PUFA		Cholest	
							
Food Group	Mean	Median	(SD)	%	order	%	order	%	order	%	order	%	order	%	order
**Beverages (incl. juices but no drinks from restgroup)**															
	**486,2**			**5,2**		**0,5**		**0,3**		**0,4**		**0,4**		**0,3**	
Water	224,2	150,0	(226.4)	0,0		0,0		0,0		0,0		0,0		0,0	
Light beverages	23,1	0,0	(90.1)	0,0		0,0		0,0		0,0		0,0		0,0	
Tea and coffee without sugar	8,2	0,0	(43.5)	0,0		0,0		0,0		0,0		0,0		0,0	
Fruit juice	172,8	150,0	(209.3)	4,5	6	0,0		0,0		0,0		0,0		0,0	
Vegetable juice	0,2	0,0	(6.0)	0,0		0,0		0,0		0,0		0,0		0,0	
Soup/bouillon	57,7	0,0	(101.7)	0,6		0,4		0,3		0,4		0,4		0,3	
**Bread and cereals**	**86,7**			**16,4**		**6,5**		**6,4**		**6,3**		**10,1**		**15,0**	
Bread/rolls/crackers/rice cakes	70,3	62,5	(46.8)	12,4	1	4,5	8	4,0	8	4,8	10	8,3	4	12,1	2
Sugared bread	7,5	0,0	(22.5)	1,7		1,5		1,9		1,1		0,8		2,9	
Breakfast cereals (ready-to-eat/hot)	8,9	0,0	(20.0)	2,3		0,5		0,5		0,4		1,0		0,0	
**Potatoes and grains**	**86,7**			**5,4**		**1,6**		**1,2**		**1,5**		**2,6**		**1,1**	
Pasta/noodles	15,4	0,0	(41.0)	1,1		0,2		0,1		0,1		0,4		0,0	
Rice	6,3	0,0	(25.5)	0,6		0,1		0,0		0,0		0,1		0,0	
Potatoes	65,0	50,0	(69.3)	3,7	7	1,4		1,1		1,4		2,0		1,1	
**Vegetables**	**66,5**			**1,1**		**0,3**		**0,3**		**0,1**		**0,5**		**0,1**	
Cooked vegetables	53,7	40,0	(60.1)	1,0		0,3		0,3		0,1		0,5		0,1	
Raw vegetables	12,8	0,0	(38.3)	0,1		0,0		0,0		0,0		0,0		0,0	
**Fruits (sweetened/unsweetened)**	**109,9**			**4,4**		**0,1**		**0,1**		**0,1**		**0,3**		**0,0**	
Fresh fruit	94,0	68,8	(102.7)	3,6	8	0,1		0,1		0,0		0,3		0,0	
Canned fruit	15,4	0,0	(45.4)	0,7		0,0		0,0		0,0		0,0		0,0	
Dried fruit	0,4	0,0	(3.7)	0,1		0,0		0,0		0,0		0,0		0,0	
Olives	0,1	0,0	(1.5)	0,0		0,0		0,0		0,0		0,0		0,0	
**Milk, milk products and calcium enriched soy milk**	**439,9**			**19,9**		**15,7**		**22,0**		**12,1**		**6,6**		**12,1**	
Milk^a^	179,0	125,0	(218.5)	6,2	4	7,1	5	10,1	2	5,4	7	1,0		5,7	8
Flavoured milk drinks (e.g. Fristi, chocolate milk,...)	188,3	145,0	(226.8)	8,9	3	4,4	9	6,2	6	3,5		1,9		3,2	9
Yoghurt	4,5	0,0	(25.3)	0,2		0,1		0,2		0,1		0,0		0,1	
Sugared or aromatised yoghurt	14,2	0,0	(46.9)	0,9		0,4		0,7		0,2		0,0		0,4	
Soy drinks	15,7	0,0	(82.5)	0,6		0,7		0,3		0,5		2,6		0,0	
Milk desserts	19,9	0,0	(56.2)	1,7		1,5		2,3		1,1		0,3		1,3	
Desserts on the basis of soy	2,3	0,0	(19.1)	0,1		0,1		0,0		0,1		0,3		0,0	
Probiotics (e.g. actimel, yakult, ...)	0,7	0,0	(7.4)	0,0		0,0		0,0		0,0		0,0		0,0	
White (fresh) cheese	15,3	0,0	(43.3)	1,4		1,5		2,2		1,3		0,4		1,4	
**Cheese**	**14,5**			**3,5**		**8,2**		**11,6**		**6,7**		**1,5**		**8,2**	
Hard cheese^b^	11,8	0,0	(22.6)	3,0		6,9	6	9,8	3	5,7	6	1,2		7,0	7
Cheese spread	2,7	0,0	(8.8)	0,5		1,2		1,8		1,0		0,3		1,3	
**Fat & oil**^**c**^	**8,6**			**3,3**		**10,5**		**8,9**		**9,3**		**20,6**		**2,4**	
Butter/margarine	8,3	6,0	(9.5)	3,1		9,9	3	8,7	5	8,3	4	19,8	1	2,4	
Oil	0,3	0,0	(1.4)	0,2		0,5		0,2		0,9		0,7		0,0	
Frying oil	0,0	0,0	(0.6)	0,0		0,1		0,0		0,1		0,1		0,0	
**Meat/poultry/fish/egg/meat alternates**	**90,3**			**13,5**		**23,2**		**18,4**		**27,3**		**22,5**		**43,3**	
Meat, game and meat products	37,2	20,0	(46.1)	6,0	5	10,6	2	9,4	4	12,9	1	6,6	6	12,5	1
Chicken/turkey	15,9	0,0	(34.7)	1,9		2,0		1,3		2,5		2,7	10	9,1	5
Fish/shellfish	8,5	0,0	(28.7)	0,9		1,1		0,5		1,1		2,9	9	3,0	10
Cold cuts (from meat poducts)	20,7	6,8	(30.2)	3,5	9	7,2	4	6,0	7	8,3	3	6,4	7	7,9	6
Cold cuts (from fish products)	0,9	0,0	(6.8)	0,2		0,4		0,1		0,5		0,7		0,4	
Eggs^d^	5,1	0,0	(18.2)	0,7		1,4		0,9		1,6		1,8		10,4	4
Meat substitutes (e.g. tofu, tempe, ...)	1,7	0,0	(11.6)	0,2		0,2		0,1		0,2		0,8		0,0	
Nuts and seeds	0,3	0,0	(3.4)	0,1		0,3		0,1		0,4		0,7		0,0	
**Restgroup (snacks & desserts)**	**201,8**			**26,8**		**32,9**		**30,6**		**35,6**		**34,0**		**16,5**	
Brioches	3,5	0,0	(17.0)	0,8		1,3		1,7		1,2		0,6		1,2	
Sweet snacks	43,6	32,0	(43.5)	11,9	2	13,4	1	16,4	1	11,8	2	9,4	3	10,8	3
Salty snacks	2,1	0,0	(9.8)	0,8		1,4		0,7		2,0		2,0		0,1	
Tea and coffee with sugar	3,2	0,0	(26.6)	0,0		0,0		0,0		0,0		0,0		0,0	
Soft drinks	97,7	0,0	(169.4)	2,7		0,0		0,0		0,0		0,0		0,0	
Salty sauces	12,5	0,0	(24.9)	1,6		3,9	10	1,7		5,3	8	6,8	5	1,4	
Cream	0,3	0,0	(2.6)	0,1		0,2		0,2		0,2		0,0		0,2	
Sweet sauces	0,1	0,0	(2.5)	0,0		0,0		0,0		0,0		0,0		0,0	
Chocolate	3,1	0,0	(9.5)	1,1		1,8		2,5	10	1,7		0,4		0,3	
Chocolate spread	9,4	0,0	(13.9)	3,5	10	6,0	7	3,4	9	7,5	5	9,8	2	0,1	
Other sweet spread (e.g. jam, honey, ...)	5,3	0,0	(11.6)	1,0		0,1		0,0		0,1		0,2		0,0	
Sugar	0,1	0,0	(0.9)	0,0		0,0		0,0		0,0		0,0		0,0	
Fried snacks	0,1	0,0	(2.6)	0,0		0,0		0,0		0,0		0,0		0,0	
French fries/croquettes	14,6	0,0	(37.7)	2,6		3,7		2,4		5,1	9	4,4	8	0,7	
Sweet desserts (e.g. ice cream, tiramisu, ...)	6,2	0,0	(23.2)	0,8		1,1		1,5		0,8		0,3		1,8	
**Miscellaneous**	**4,2**			**0,5**		**0,5**		**0,3**		**0,6**		**0,8**		**0,9**	
Pizza & quiches	2,2	0,0	(17.8)	0,3		0,3		0,2		0,4		0,5		0,6	
Other miscellaneous^e^	2,0	0,0	(21.3)	0,2		0,2		0,1		0,2		0,3		0,3	

The contributions of each food group are expressed in percentage of daily energy and nutrient intakes.

^a ^Includes cow's milk and goat's milk

^b ^Excludes cream cheese

^c ^Includes lard/animal fats and regular/low-fat/fat-free versions of cream cheese/sour cream/half-and-half

^d ^includes only eggs reported separately and eggs included in disaggregated food mixtures

^e ^includes foods or components with negligible contributions to total nutrient intakes that could not be categorized in the above food groups (e.g. herbs and spices/monosodium glutamate/starch/plain gelatin/artificial sweeteners/pectin/cocoa powder/etc.)

^f ^These mean food group intakes are only rough estimates calculated from the raw data on which these nutrient contributions are based, without adjustment for within person variability. The high number of non-consumers in some of the food groups hindered the adjustment for within-individual variability.

**Table 3:** Contribution from all food groups to protein, carbohydrates and water (n = 696)

	Nutrients and dietary constituents												
	**Food intake**^**f**^			**Protein**		**CH**		**Simp. CH**		**Comp. CH**		**Water**	
						
**Food Group**	**Mean**	**Median**	**(SD)**	**%**	**order**	**%**	**order**	**%**	**order**	**%**	**order**	**%**	**order**

**Beverages (incl. juices but no drinks from restgroup)**	**486,2**			**2,0**		**8,8**		**14,8**		**1,2**		**36,6**	
Water	224,2	150,0	(226.4)	0,0		0,0		0,0		0,0		17,7	1
Light beverages	23,1	0,0	(90.1)	0,0		0,1		0,1		0,0		1,8	
Tea and coffee without sugar	8,2	0,0	(43.5)	0,0		0,0		0,0		0,0		0,6	
Fruit juice	172,8	150,0	(209.3)	1,2		8,1	4	14,4	2	0,0		12,1	4
Vegetable juice	0,2	0,0	(6.0)	0,0		0,0		0,0		0,0		0,0	
Soup/bouillon	57,7	0,0	(101.7)	0,7		0,7		0,3		1,2		4,3	7
**Bread and cereals**	**86,7**			**12,9**		**23,2**		**3,7**		**48,7**		**2,0**	
Bread/rolls/crackers/rice cakes	70,3	62,5	(46.8)	10,3	4	17,7	1	1,1		39,3	1	1,8	10
Sugared bread	7,5	0,0	(22.5)	1,4		1,8		0,3		3,8	6	0,2	
Breakfast cereals (ready-to-eat/hot)	8,9	0,0	(20.0)	1,1		3,7	9	2,3		5,6	4	0,0	
**Potatoes and grains**	**86,7**			**3,9**		**8,0**		**1,0**		**17,4**		**5,1**	
Pasta/noodles	15,4	0,0	(41.0)	0,9		1,6		0,3		3,4	7	0,9	
Rice	6,3	0,0	(25.5)	0,4		1,1		0,0		2,5	8	0,3	
Potatoes	65,0	50,0	(69.3)	2,6	10	5,4	6	0,7		11,4	3	3,9	8
**Vegetables**	**66,5**			**2,0**		**1,4**		**1,8**		**0,8**		**4,8**	
Cooked vegetables	53,7	40,0	(60.1)	1,8		1,1		1,4		0,8		3,8	9
Raw vegetables	12,8	0,0	(38.3)	0,2		0,2		0,3		0,1		1,0	
**Fruits (sweetened/unsweetened)**	**109,9**			**1,3**		**7,8**		**13,2**		**0,9**		**7,2**	
Fresh fruit	94,0	68,8	(102.7)	1,2		6,4	5	10,7	4 10	0,9		6,2	6
Canned fruit	15,4	0,0	(45.4)	0,1		1,3		2,3		0,0		1,0	
Dried fruit	0,4	0,0	(3.7)	0,0		0,1		0,3		0,0		0,0	
Olives	0,1	0,0	(1.5)	0,0		0,0		0,0		0,0		0,0	
**Milk, milk products and calcium enriched soy milk**	**439,9**			**27,3**		**20,4**		**33,7**		**2,8**		**29,5**	
Milk^a^	179,0	125,0	(218.5)	11,3	2	4,3	8	7,6	6	0,0		12,6	2
Flavoured milk drinks (e.g. Fristi, chocolate milk,...)	188,3	145,0	(226.8)	10,5	3	11,1	3	18,4	1	1,7	9	12,4	3
Yoghurt	4,5	0,0	(25.3)	0,4		0,1		0,3		0,0		0,3	
Sugared or aromatised yoghurt	14,2	0,0	(46.9)	1,0		1,1		2,0		0,0		0,9	
Soy drinks	15,7	0,0	(82.5)	1,0		0,4		0,5		0,1		1,1	
Milk desserts	19,9	0,0	(56.2)	1,4		1,9		2,6	9	0,9		1,1	
Desserts on the basis of soy	2,3	0,0	(19.1)	0,1		0,2		0,2		0,1		0,2	
Probiotics (e.g. actimel, yakult, ...)	0,7	0,0	(7.4)	0,0		0,1		0,1		0,0		0,0	
White (fresh) cheese	15,3	0,0	(43.3)	1,6		1,2		1,9		0,0		0,9	
**Cheese**	**14,5**			**5,9**		**0,1**		**0,1**		**0,0**		**0,5**	
Hard cheese^b^	11,8	0,0	(22.6)	5,2	7	0,0		0,0		0,0		0,4	
Cheese spread	2,7	0,0	(8.8)	0,7		0,0		0,1		0,0		0,1	
**Fat & oil**^**c**^	**8,6**			**0,1**		**0,0**		**0,0**		**0,0**		**0,3**	
Butter/margarine	8,3	6,0	(9.5)	0,1		0,0		0,0		0,0		0,3	
Oil	0,3	0,0	(1.4)	0,0		0,0		0,0		0,0		0,0	
Frying oil	0,0	0,0	(0.6)	0,0		0,0		0,0		0,0		0,0	
**Meat/poultry/fish/egg/meat alternates**	**90,3**			**35,1**		**1,3**		**0,2**		**2,7**		**4,3**	
Meat, game and meat products	37,2	20,0	(46.1)	15,6	1	0,7		0,0		1,6	10	1,7	
Chicken/turkey	15,9	0,0	(34.7)	8,0	5	0,1		0,0		0,1		0,8	
Fish/shellfish	8,5	0,0	(28.7)	3,0	9	0,2		0,0		0,4		0,5	
Cold cuts (from meat poducts)	20,7	6,8	(30.2)	6,5	6	0,2		0,1		0,3		1,0	
Cold cuts (from fish products)	0,9	0,0	(6.8)	0,3		0,0		0,0		0,0		0,0	
Eggs^d^	5,1	0,0	(18.2)	1,3		0,0		0,0		0,0		0,3	
Meat substitutes (e.g. tofu, tempe, ...)	1,7	0,0	(11.6)	0,3		0,1		0,0		0,2		0,1	
Nuts and seeds	0,3	0,0	(3.4)	0,1		0,0		0,0		0,0		0,0	
**Restgroup (snacks & desserts)**	**201,8**			**9,0**		**28,4**		**31,4**		**24,4**		**9,6**	
Brioches	3,5	0,0	(17.0)	0,4		0,7		0,3		1,1		0,1	
Sweet snacks	43,6	32,0	(43.5)	5,0	8	13,0	2	11,2	3	15,1	2	0,6	
Salty snacks	2,1	0,0	(9.8)	0,2		0,6		0,0		1,2		0,0	
Tea and coffee with sugar	3,2	0,0	(26.6)	0,0		0,1		0,1		0,0		0,2	
Soft drinks	97,7	0,0	(169.4)	0,0		5,0	7	8,9	5	0,0		6,9	5
Salty sauces	12,5	0,0	(24.9)	0,6		0,6		0,7		0,5		0,7	
Cream	0,3	0,0	(2.6)	0,0		0,0		0,0		0,0		0,0	
Sweet sauces	0,1	0,0	(2.5)	0,0		0,0		0,1		0,0		0,0	
Chocolate	3,1	0,0	(9.5)	0,4		0,8		1,5		0,0		0,0	
Chocolate spread	9,4	0,0	(13.9)	1,0		2,8	10	4,6	7	0,5		0,0	
Other sweet spread (e.g. jam, honey, ...)	5,3	0,0	(11.6)	0,1		1,8		2,9	8	0,4		0,1	
Sugar	0,1	0,0	(0.9)	0,0		0,0		0,1		0,0		0,0	
Fried snacks	0,1	0,0	(2.6)	0,0		0,0		0,0		0,0		0,0	
French fries/croquettes	14,6	0,0	(37.7)	0,9		2,4		0,1		5,4	5	0,6	
Sweet desserts (e.g. ice cream, tiramisu, ...)	6,2	0,0	(23.2)	0,4		0,7		1,1		0,2		0,3	
**Miscellaneous**	**4,2**			**0,6**		**0,4**		**0,2**		**0,7**		**0,2**	
Pizza & quiches	2,2	0,0	(17.8)	0,4		0,3		0,1		0,5		0,1	
Other miscellaneous^e^	2,0	0,0	(21.3)	0,2		0,1		0,1		0,2		0,1	

The contributions of each food group are expressed in percentage of daily nutrient intakes.

^a^Includes cow's milk and goat's milk

^b^Excludes cream cheese

^c^Includes lard/animal fats and regular/low-fat/fat-free versions of cream cheese/sour cream/half-and-half

^d^includes only eggs reported separately and eggs included in disaggregated food mixtures

^e^includes foods or components with negligible contributions to total nutrient intakes that could not be categorized in the above food groups (e.g. herbs and spices/monosodium glutamate/starch/plain gelatin/artificial sweeteners/pectin/cocoa powder/etc.)

^f^These mean food group intakes are only rough estimates calculated from the raw data on which these nutrient contributions are based, without adjustment for within person variability. The high number of non-consumers in some of the food groups hindered the adjustment for within-individual variability.

Simp. CH, simple carbohydrates; Comp. CH, complex carbohydrates

The Ethical Committee of the Ghent University Hospital (Belgium) granted ethical approval for the study. Signed informed consent was obtained from the parents of all the children participating in the Flanders preschool dietary survey.

### Statistical analyses

Statistical analyses were performed with the Statistical Package for the Social Sciences for Windows version 14 (SPSS Inc., Chicago, IL, USA). The population proportion formula was used to determine the percentage contribution of each of the 57 food groups to the intake of each dietary component. This was done by summing the amount of the component provided by the food for all individuals divided by the total intake of that component from all foods for the entire study population [7,16,17].

Since the average of a small number of days does not adequately reflect an individual's usual intake, statistical modelling of dietary intakes is needed [18]. In order to correct for day-to-day variability in the 3d EDR, mean and median 'usual' intakes of the population and the proportion below or above defined cut-offs were calculated using statistical modelling (the NUSSER method, developed at Iowa State University) [19,20]. When using consecutive days, at least three days are required to estimate usual dietary intakes by means of the NUSSER method [19,20]. The programme used to calculate usual intakes was the Software for Intake Distribution Estimation (C-side) [21]. The proportion of the variance on nutrient intakes explained by schools and classes was low (< 5%) in the present study, so clustering effects were not addressed during analysis. Because of the high number of non-consumers in some of the detailed food(group)s, adjusted mean intakes could not be calculated for those food(group)s. However, to give an impression of the magnitude of intakes of the different food(group)s in order to help interpreting the contributions, unadjusted mean and median intakes were added to the tables (tables 2 and 3). The Belgian recommended dietary allowances were used as reference values for the nutrient intakes [22], age-specific Food Based Dietary Guidelines (FBDG) were used as reference for food(group) intakes [23].

## Results

### Energy and macronutrients

Tables 2 &3 show that bread, sweet snacks (cakes/cookies/candy...), milk, and flavoured milk drinks belong to the top ten sources of energy, fat, protein, and carbohydrates. Meat products (including cold cuts) are also among the top ten sources of energy, fat, and protein, but not of carbohydrates. Butter and margarine are the main source of PUFA (20%), while meat and sweet snacks are the main source of MUFA (13% and 12%, respectively). Sweet snacks and milk (including flavoured milk drinks) are the main source of SFA (16%), followed by hard cheese (10%).

Meat is the main contributor to cholesterol intake (13%), followed by bread (12%) and sweet snacks (11%). Flavoured milk drinks and fruit juice are the main sources of simple carbohydrates (18% and 14%, respectively), followed by sweet snacks (11%). Bread is the main contributor to complex carbohydrates (39%). Water, milk, and flavoured milk drinks give the highest contribution to total water intake (18%, 13% and 12%, respectively).

### Food sources and nutrient and food adequacy

Table 4 presents a brief summary of nutrient and food intakes which are under-consumed by more than 30% of the children. Nutrients that were importantly under-consumed are total fat, PUFA, MUFA and water. From tables 2 and 3 it could be concluded that the main food sources contributing to those nutrients are respectively sweet snacks for total fat, margarine for PUFA, meat for MUFA and mineral or tap water for water. Foods that were underconsumed by more than 30% of the children were beverages (not from residual group), bread and cereal, vegetables, fruit, milk and spreadable fats (table 4).

**Table 4:** Mean and median intakes of nutrients and foods and the % of the population that had intakes below the minimum recommendation, calculated with adjustment for within-individual variability^a^

Nutrient	Age	Reference values	Mean (SD)	Median (SE)	**% < LL/AMDR**_**LL **_**(SE)**
Total fat*	< 4 years	35 - 40%	29,8 (4.5)	29,8 (0.5)	77 (5.0)
	≥ 4 years	30 - 35%	29,9 (3.6)	29,8 (0.3)	52 (3.0)
Monounsaturated	< 4 years	> 12%	10,6 (1-7)	10,6 (0-2)	80 (6.0)
fatty acids*	≥ 4 years		10,7 (1.4)	10,7 (0.1)	82 (0.4)
Polyunsaturated	< 4 years	> 8%	4,4 (1-2)	4,2 (0-1)	99 (1.0)
fatty acids*	≥ 4 years		4,5 (1.1)	4,3 (0.1)	100 -
Water	< 4 years	75 - 100 ml/kg/day^‡^	77,8 (16-9)	76,0 (1-6)	47,0 (4.0)
	≥ 4 years		64,6 (14.8)	63,5 (0.9)	78,0 (3.0)

**Food group**	**Age**	**FBDG**	**Mean (SD)**	**Median (SE)**	**% < FBDG_LL_(SE)**

Beverages (not from restgroup)^¶^	< 4 years	500-1000 ml	504,8 (197.5)	487,0 (17.6)	99 (0.7)
	≥ 4 years	1500 ml	540,7 (241.5)	514,0 (14.0)	96 (0.9)
Bread & cereais	< 4 years	30-100 g	97,1 (39.6)	91,0 (3.2)	49 (3.6)
	≥ 4 years	100-150 g	94,7 (30.7)	92,0 (2.1)	47 (2.3)
Vegetables	< 4 years	50-100 g	65,8 (19.7)	65,0 (1.8)	95 (1.6)
	≥ 4 years	100-150 g	75,1 (30.3)	72,0 (1.8)	82 (1.8)
Fruit	< 4 years	100-200 g	118,2 (57.8)	116,0 (5.2)	57 (3.5)
	≥ 4 years		112,1 (59.5)	105,0 (3.5)	63 (2.2)
Milk	< 4 years	500 ml	514,0 (227.0)	504,0 (20.3)	49 (3.6)
	≥ 4 years		446,4 (197.9)	428,0 (11.5)	64 (2.2)
Spreadable fat (No cooking fat)	< 4 years	15-25 2	4,9 (5.5)	3,3 (0.5)	94 (1.7)
	≥ 4 years		5,3 (5.6)	3,6 (0.3)	94 (1.1)

^a ^Only nutrients and food groups for which the percentage of childre n with low inta kes was > 30% are presented.

* Percentage of total energy intake supplied by the respective nutrient and its recommendation expressed as acceptable macronutrient distribution ranges (AMDR).

¶ All drinks not included in the restgroup and no milkproducts

‡ Acceptable range (AR)

Lower Level (LL)

Food Based Dietary Guidelines (FBDG)

n (< 4 years old) 197

n (≥ 4 years old) 465

In table 5, a brief summary of nutrient and food intakes which are overconsumed by more than 30% of the children is presented. Nutrients that were importantly over-consumed are protein, SFA and simple carbohydrates. From tables 2 and 3 it could be concluded that the main food sources contributing to those nutrients are respectively meat and milk (for protein), sweet snacks (for SFA and simple carbohydrates) and flavoured milk drinks and fruit juice (for simple carbohydrates). Foods that were over-consumed by more than 30% of the children were all foods from the residual group (snacks/desserts, sugared drinks, fried potatoes, sauces and sweet spreads).

**Table 5:** Mean and median intakes of nutrients and foods and the % of the population that had intakes above the upper levels, calculated with adjustment for within-individual variability^a^

Nutrient	Age	Reference values	Mean (SD)	Median (SE)	**% > UL/AMDR**_**UL **_**(SE)**
Protein*	< 4 years	10 - 15%	16,4 (2.1)	16,3 (0.2)	75 (5.0)
	≥ 4 years		15,3 (2.1)	15,2 (0.2)	53 (3.0)
Saturated fatty	< 4 years	8 - 12%	13,4 (2.7)	13,3 (0.3)	69 (4.0)
acids*	≥ 4 years		13,4 (1.9)	13,4 (0.2)	77 (4.0)
Simple	< 4 years	< 15%^£^	30,7 (5.3)	30,6 (0.6)	100 -
carbohydrates*	≥ 4 years		31,4 (5.2)	31,3 (0.4)	100 -

**Food group**	**Age**	**FBDG**	**Mean (SD)**	**Median (SE)**	**%> FBDG**_**UL **_**(SE)**

Restgroup (snacks/desserts)†	< 4 years	restricted	46,8 (13.3)	45,1 (1.2)	36 (3.4)
	≥ 4 years		53,7 (16.4)	52,0 (1.0)	55 (2.3)
Restgroup (sugared drinks)‡	< 4 years	restricted	89,9 (106.2)	56,0 (9.5)	35 (3.4)
	≥ 4 years		123,7 (131.5)	72,0 (7.6)	40 (2.3)
Restgroup (fried potatoes)μ	< 4 years	restricted	12,6 (9.3)	11,4 (0.8)	36 (3.4)
	≥ 4 years		14,2 (6.0)	13,5 (0.3)	38 (2.3)
Restgroup (sauces)μ	< 4 years	restricted	12,5 (5.2)	13,1 (0.5)	34 (3.4)
	≥ 4 years		12,9 (4.5)	13,2 (0.3)	32 (2.2)
Restgroup (sweet spreads)^μ^	< 4 years	restricted	14,4 (8.8)	12,4 (0.8)	38 (3.5)
	≥ 4 years		14,4 (8.7)	13,2 (0.5)	41 (2.3)

a Only nutrients and food groups for which the percentage of children with excessive intakes was > 30% are presented.

* Percentage of total energy intake supplied by the respective nutrient and its recommendation expressed as acceptable macronutrient distribution ranges (AMDR).

† Sweet deserts (e.g. ice cream, tiramisu), sweet snacks, salty snacks (e.g. chips), chocolate, and brioches. Although the recommendation is to limit these food products, the percentage given in the column > FBDG_UL _are children consuming more than 50 g/d of these snacks.

‡ Sugared drinks (e.g. tea with sugar added) and softdrinks, but no fruit juices. Although the recommendation is to limit these food products, the percentage given in the column > FBDG_UL _are children consuming more than 100 ml/d of these sugared drinks.

^μ ^Although the recommendation is to limit these food products, the percentage given in the column > FBDG_UL _are children consuming more than 15 g/d of these sauces.

£No national recommendations are available for simple carbohydrates. Therefore, intakes were compared with a reference value of 15 precent of total energy intake.

Upper level (UL)

Food Based Dietary Guidelines (FBDG)

n (< 4 years old) 197, n (≥4 years old) 465

According to the Flemish FBDG, fruit juice and flavoured milk drinks belong to the food products that can be used with moderation and therefore do not belong to the food group of sugared drinks which are overconsumed.

Results in table 4 &5 are split for children below and above four years of age enabling comparison of intakes with nutrient recommendations and FBDGs.

## Discussion

Understanding the dietary intake of a population requires the investigation of the intake of individual nutrients, but also of foods and contributions of foods to nutrient intakes. Since this is the first study to provide a detailed list of principal food sources of energy and macronutrient intakes in Flemish preschoolers, it can be used to formulate suggestions in order to increase the compliance of nutrient and food intakes with the current recommendations.

### Main results

Bread, sweet snacks, flavoured milk drinks, milk, and meat products were the top five sources of energy intake among Flemish preschoolers. However, for the bread group, it is noteworthy that this group also contains rolls and croissants, which were often consumed by preschoolers and partly responsible for the important contribution of the bread group to energy, SFA and cholesterol intakes. Also, sweet snacks were major sources of total fat and SFA, and the third important source of simple carbohydrate intakes after flavoured milk drinks and fruit juices.

While spreadable margarine for bread was underconsumed in comparison with the FBDG, it was still the main source of PUFA intakes. Although spreadable fat is an item that might be more prone to underreporting in diaries, the results from the FFQ confirmed that more than one-third of the children never consumed spreadable fat on their bread [4].

### Food sources and nutrient and food adequacy: a basis for evaluating dietary guidelines

When comparing tables 4 &5, while taking into account the main food sources for those nutrients discussed above (tables 2 &3), interesting recommendations/guidelines could be formulated in order to pursue the designated nutritional goals. Insufficient nutrient intakes in Flemish preschoolers, should be increased by enhancing the intake of food(group)s that highly contribute to these particular nutrients, but are underconsumed in comparison with the FBDG. In the same way, excessive nutrient intakes should be decreased by reducing the consumption of food(group)s with high contributions to those particular nutrients, though overconsumed in this population.

When looking at the food groups that are underconsumed in comparison with the FBDG (table 4) and taking into account the contributions of these foods to the nutrients being inadequately consumed, it can be concluded that higher intakes of non-sugared beverages (mainly water) could contribute importantly to the increase in water intake. Although an increase in milk intake can contribute to higher fluid intakes, it would also increase the intake of many other nutrients like SFA. Enhancing the daily amount of spreadable margarine for preschoolers' bread and increasing fish intake could contribute importantly to the increase of PUFA intakes.

When looking at the food groups that are overconsumed in comparison with the FBDG (table 5) and taking into account the contributions of these foods to nutrients that exceed the upper intake level, it can be concluded that lower intakes of sweet snacks would lower importantly the SFA and simple carbohydrate intakes among Flemish preschoolers. Also, a decrease in the consumption of sugared drinks and fruit juices would significantly decrease simple carbohydrate intakes. Replacement of flavoured milk drinks by natural milk should for instance be encouraged. The current consumptions of sweet spreads (like jam and chocolate spread) are only a case of concern with regard to the intake of simple sugars, though their contribution to simple sugars is still lower than that from flavoured milk drinks, fruit juices, soft drinks, and sweet snacks. Furthermore, it could be concluded from tables 2 and 3 that a lower consumption of fat-rich cold cuts could help to decrease SFA intakes, given its high contribution and its high consumption in comparison with other products from the 'meat group'. At last, a lower consumption of hard cheese or replacement by low-fat types or cottage cheese could also reduce SFA intakes.

### Methodological considerations

Some limitations should be considered when interpreting or using these results. First of all, it should be underlined that the information collected from these Flemish preschoolers relies upon parents and/or other proxies' capabilities of recall. However, several steps were undertaken in this study to increase the validity of the information (e.g. school staff was involved in the reporting of snacks and lunches consumed during school-time, and great efforts were done to motivate the parents). Nevertheless, it should be noted that the underrepresentation of lower educated parents could result in participation bias warranting caution in generalisation of the current findings.

Second, decisions about food grouping were based on the food groups in our Flemish FBDG and on the judgement of the investigators, which might have implications for the findings. For example, rolls and croissants were classified under bread products according to the main food group classification, however, within the bread food group it is considered as a food item from the residual group. Also, decisions regarding the disaggregating of mixed foods might have consequences for the present results. For example, disaggregating pizza would have given a more realistic estimate of how cheese contributes to nutrient intakes but does not allow for knowing how pizza itself contributes.

Since all days of the week were included in the study, the effect of day of the week could be removed. Unfortunately, it was impossible to correct for seasonal variations, because data was collected during autumn and wintertime. However, in the Belgian National Food Consumption Survey performed in 2004, it was concluded that seasonal variation was limited for nutrient intakes [15]. A possible explanation might be the widespread availability of most foods all year round.

Differences in methodology and ways of grouping foods hamper comparisons with other studies. However, an essential finding that was comparable with other studies investigating the major sources of energy and nutrient intakes among children was the important contribution of fortified foods to children's diets [7,24].

Finally, caution is necessary when interpreting these results since food composition data do not consider bio-availability of nutrient sources.

### Suggestions for future dietary guidelines and policies

From previous studies investigating nutrient and food group adequacies in Flemish preschoolers [3,4], it could be concluded that preschoolers in Flanders should be recommended a different dietary pattern in order to pursue the designated nutritional goals. More specifically, fibre, iron and vitamin D intakes were well below recommendations, while sodium and saturated fatty acid intakes exceeded tolerable upper intake levels [3]. Furthermore, the percentage of children complying with FBDGs was for most food groups extremely low (ranging from 4% for fluids and vegetables up to 99% for potato intakes) [4]. The current study investigating nutrient sources, additionally revealed some important recommendations in order to increase nutrient and food intake compliances with the current recommendations. However, this study also raised concern about some of the current dietary guidelines, which are discussed below.

Given the fact that whole fat milk is still recommended for children younger than four years old and considering the high SFA contributions of milk products, it could be suggested to replace whole fat milk by half-fat varieties in FBDG for preschoolers. Supply of fat-soluble vitamins could then be compensated by use of margarine on children's bread, which is currently being underconsumed in comparison with the recommendations and which, at the same time, would contribute to higher PUFA intakes. Accordingly, nutritional policies targeted at replacing whole fat milk with another low-fat (e.g. semi-skimmed) variety, should be implemented as key strategy for achieving recommended SFA intake levels in this age group [25,26].

Furthermore, it should be noted that excessive consumption of fruit juice in infants and children has typically been related to carbohydrate malabsorption [27], dental caries, and gastrointestinal symptoms such as bloating, diarrhoea, and cramping [28]. More recently, fruit juices have also been blamed as possible contributors to the current childhood obesity epidemic in the US where fruit juices were seen as healthy and convenient replacements of fresh fruits [29]. Therefore, it should be stressed to parents and caregivers that fruit juices should not be used as a replacement for fresh fruits. More importantly, FBDG compilers should re-evaluate fruit juice's grouping as a food that can be consumed with moderation. In the present study, fruit juice consumption is the second highest contributor of simple carbohydrate intakes in preschoolers, therefore, fruit juice should be categorised as a food item from the residual group.

The high intake of fortified biscuits seen in Flemish preschoolers results in an increase of energy, SFA, and simple carbohydrates, all counteradvised in the prevention of certain chronic diseases. Furthermore, a 'habit' of eating biscuits/cakes that is formed during childhood is likely to continue into adulthood [30,31]. Therefore, children should be recommended to replace sweet snacks by more healthy foods like bread with margarine (combined with low-fat cold cuts or cottage cheese), fruits or certain vegetables (e.g. baby carrots) in order to decrease SFA and simple carbohydrate intakes.

Since this study includes the first comprehensive examination of food sources of nutrients in Flemish preschoolers, it can be used for establishing/revising guidelines for Flemish preschoolers. Though, as the food supply changes, these data will need to be continually updated.

## Conclusion

Some guidelines to improve Flemish preschoolers' dietary habits could be derived from this study. The intake of sweet snacks and sugar rich drinks (like soft drinks or fruit juices) should be discouraged, while the consumption of fruits, vegetables, water, bread, and margarine on bread should be encouraged. Replacement of fat-rich foods from the SFA-rich food groups 'meat products' and 'dairy' by lean or low-fat alternatives should be recommended. Furthermore it should be emphasised that selection of a variety of foods is the best way to provide a desirable balance, without excessive intakes of macronutrients, micronutrients, and other components of foods, and should be recommended above the consumption of fortified foods.

## Competing interests

The authors declare that there are no competing interests. Carine Vereecken is postdoctoral researcher funded by the FWO-Flanders.

## Authors' contributions

IH, YL and WDK were responsible for the analyses. WDK and IH drafted the manuscript. All other authors helped in the evaluation of the results and commented on the manuscript. Moreover, IH and SDH were reponsible for the study protocol and the fieldwork. All authors have read and approved the manuscript as submitted.
